# SwissTargetPrediction: a web server for target prediction of bioactive small molecules

**DOI:** 10.1093/nar/gku293

**Published:** 2014-05-03

**Authors:** David Gfeller, Aurélien Grosdidier, Matthias Wirth, Antoine Daina, Olivier Michielin, Vincent Zoete

**Affiliations:** 1 Swiss Institute of Bioinformatics (SIB), Quartier Sorge, Bâtiment Génopode, CH-1015 Lausanne, Switzerland; 2 Ludwig Institute for Cancer Research, Centre Hospitalier Universitaire Vaudois, Lausanne, Switzerland; 3 Oncology Department, Centre Hospitalier Universitaire Vaudois, Lausanne, Switzerland

## Abstract

Bioactive small molecules, such as drugs or metabolites, bind to proteins or other macro-molecular targets to modulate their activity, which in turn results in the observed phenotypic effects. For this reason, mapping the targets of bioactive small molecules is a key step toward unraveling the molecular mechanisms underlying their bioactivity and predicting potential side effects or cross-reactivity. Recently, large datasets of protein–small molecule interactions have become available, providing a unique source of information for the development of knowledge-based approaches to computationally identify new targets for uncharacterized molecules or secondary targets for known molecules. Here, we introduce SwissTargetPrediction, a web server to accurately predict the targets of bioactive molecules based on a combination of 2D and 3D similarity measures with known ligands. Predictions can be carried out in five different organisms, and mapping predictions by homology within and between different species is enabled for close paralogs and orthologs. SwissTargetPrediction is accessible free of charge and without login requirement at http://www.swisstargetprediction.ch.

## INTRODUCTION

Molecular insight into the mode of action of bioactive small molecules is key to understanding observed phenotypes, predicting potential side effects or cross-reactivity and optimizing existing compounds (1–3). In particular, mapping their targets is a crucial step toward providing a rational understanding of small molecule's bioactivity. For these reasons, high-throughput reverse screening of chemical compounds against arrays of protein targets has become an integral part of drug discovery pipelines (4). As a result, for many proteins such as specific kinases or phosphatases, hundreds of small molecule ligands have been identified. Such large screening initiatives have also provided unique insights into the specificity and pharmacology of protein families (1,5). Recently, these data have been collected in several public databases, like ChEMBL (6) or PubChem (7) storing information on bioactivities, or ZINC (8) containing information on commercially available compounds. These can be mined automatically to retrieve specific information for a large number of molecules.

However, molecular targets still remain unknown in several cases. For instance, phenotypic assays indicate whether a molecule is active or not, without necessarily providing direct information on its actual molecular targets (9–11). Moreover, for most molecules, experiments have been performed with a limited set of targets, such as kinases or G protein-coupled receptors, and possible off-target effects have been rarely tested for. Finally, new molecules being developed for specific purposes may have several targets that are typically not known in advance. For instance, a recent study on a set of 802 drugs and interaction data assembled from seven different databases has shown that known drugs have on average six molecular targets on which they exhibit activity (12). Identifying these secondary targets is crucial. First, it can indicate possible adverse side effects that might arise when using the molecule, thereby decreasing the attrition rate in clinical trials due to toxicity (13,14). Second, it provides ways of repositioning (or repurposing) molecules for new applications. This has become a central theme in pharmaceutical research in view of the difficulty to launch new chemical entities. In particular, it is increasingly being recognized that several compounds traditionally used for one given application may actually show potent activity in other therapeutic settings (2,15,16).

Computational predictions play an important role in narrowing down the set of potential targets and suggesting secondary targets for known molecules (13,15). In particular, the large amount of information collected on protein–small molecule interactions in the last few years has enabled researchers to develop ligand-based approaches for target prediction (1,17–20). With SwissTargetPrediction, our goal is to provide a user-friendly web interface for a knowledge-based algorithm, recently developed in our group (18), to predict the targets of bioactive small molecules. Compared to other existing approaches, SwissTargetPrediction has several distinctive features. First, it enables combining both 2D and 3D similarity measures with known ligands. Second, it provides results in five different species. Third, it allows users to map predictions between and within organisms based on target homology.

## THE SWISSTARGETPREDICTION METHOD AND DATASET

SwissTargetPrediction is based on the observation that similar bioactive molecules are more likely to share similar targets (1,21). Therefore, the targets of a molecule can be predicted by identifying proteins with known ligands that are highly similar to the query molecule. In this ligand-based strategy, a major challenge is to accurately identify and quantify similarity between the query molecule and the known ligands. Early approaches have focused on determining chemical similarity by using molecular fingerprints (22) (sometimes called 2D similarity). While compounds exhibiting a high similarity under these measures clearly have an increased likelihood for interactions with similar targets, the biophysics of molecular recognition suggests that similarity in ligand shape or electrostatic potential distribution could also lead to a similar effect (23). Therefore, 3D structural similarity measures have been developed to assess similarity between molecules (24–29). Recently, we have shown that combining 2D and 3D similarity measures significantly increases the target prediction accuracy, especially if the query molecule is new and does not belong to an already well-studied chemical series (18). In SwissTargetPrediction, both 2D similarity and 3D similarity values are computed against a set of known ligands. For 2D similarity, we use FP2 fingerprints to describe molecules, as implemented in OpenBabel version 2.2.0. The similarity between two molecules is quantified with the Tanimoto coefficient (which corresponds to the number of shared fingerprint patterns divided by the total number of fingerprint patterns describing the two molecules). For 3D similarity, we first generate 20 different conformations of each molecule (see Supplementary Materials). From these different conformations, 20 Electroshape vectors, which consist of 18-dimensional real vectors (27), are computed. The Manhattan distance $\left( {d = \sum\limits_{s = 1}^{18} {\left| {x_s - y_s } \right|} } \right)$ is used to compare vectors (*x* and *y*) describing two different molecules. The final 3D similarity value between molecules *i* and *j* is computed as $1/\left( {1 + \frac{1}{{18}}d_{ij} } \right)$, where *d_ij_* is the smallest Manhattan distance among the 20×20 distances calculated over all possible conformations of each molecule (see also Supplementary Materials). The final score of a target corresponds to a combination of similarity measures based on a logistic regression of the similarity values, with the most similar ligands using both 2D and 3D similarity measures (see Supplementary Materials and (18)). Coefficients of the logistic regression for each molecule size are listed in Supplementary Table S1. Target scores range therefore between 0 and 1, with the largest possible value being reached if the query molecule is a known ligand of the target. These scores are used to rank predicted targets. A probability has been derived from this score to assess the likelihood of the predictions to be correct. These probability values correspond to the average precision (i.e. number of true-positives divided by the total number of predicted targets at different thresholds) obtained in a leave-one-out cross-validation study over our training set (see Supplementary Materials). As it is based on cross-validation, they may suffer from internal biases in our training data (e.g. presence of large congeneric series of similar molecules) and if a new query molecule without related molecules in our database is tested, they may slightly overestimate the prediction accuracy. For this reason, we stress that these probabilities are primarily used to rank targets predicted to bind to a given small molecule. In particular, they should not be used to compare predictions obtained with different molecules.

The set of protein–ligand interactions was retrieved from the ChEMBL database version 16 (6) using stringent criteria to remove ambiguous cases. First, only interactions involving single proteins or protein complexes as well as ligands with less than 80 heavy atoms were considered. Second, selected interactions had to be annotated as direct binding (‘assay_type’ = ‘B’) with an activity (K_i_, K_d_, IC_50_ or EC_50_) lower than 10 μM in all assays. Interactions were retrieved in five organisms (human, mouse, rat, cow and horse). In total, our dataset consists of 280 381 small molecules interacting with 2686 targets, with the majority of targets (66%) found in human (see Table 1).

**Table 1.** Number of targets in each organism

Organisms	Number of targets	Number of targets including homology-based predictions
*Homo sapiens*	1768	2547
*Mus musculus*	342	2345
*Rattus norvegicus*	469	2657
*Bos taurus*	104	2272
*Equus caballus*	3	2367
Total	2686	12 188

The first column shows the number of targets with experimental data. The second column shows the number of targets when including homology-based predictions.

## THE SWISSTARGETPREDICTION WEB INTERFACE

SwissTargetPrediction provides an intuitive interface to predict small molecule protein targets (see also Supplementary Figure S1). Query molecules can be inputted either as SMILES, or drawn in 2D using the javascript-based molecular editor of ChemAxon (http://www.chemaxon.com). The SMILES input field and the 2D interface are automatically synchronized. The organism in which predictions should be made can be selected. The current version of SwissTargetPrediction allows users to choose between five organisms: human, mouse, rat, cow and horse, the default being human (see Supplementary Figure S1). Once a molecule has been provided, either by SMILES or by drawing, and an organism has been chosen, the ‘Submit’ button becomes clickable and calculations can start. The SMILES is first checked to ensure that it corresponds to a valid chemical structure. If true, the similarity (both 2D and 3D) between the query molecule and all ligands in our database is computed and the score of each target is derived from the combined 2D and 3D similarity values with the most similar ligands (see Supplementary Materials).

**Figure 1.**
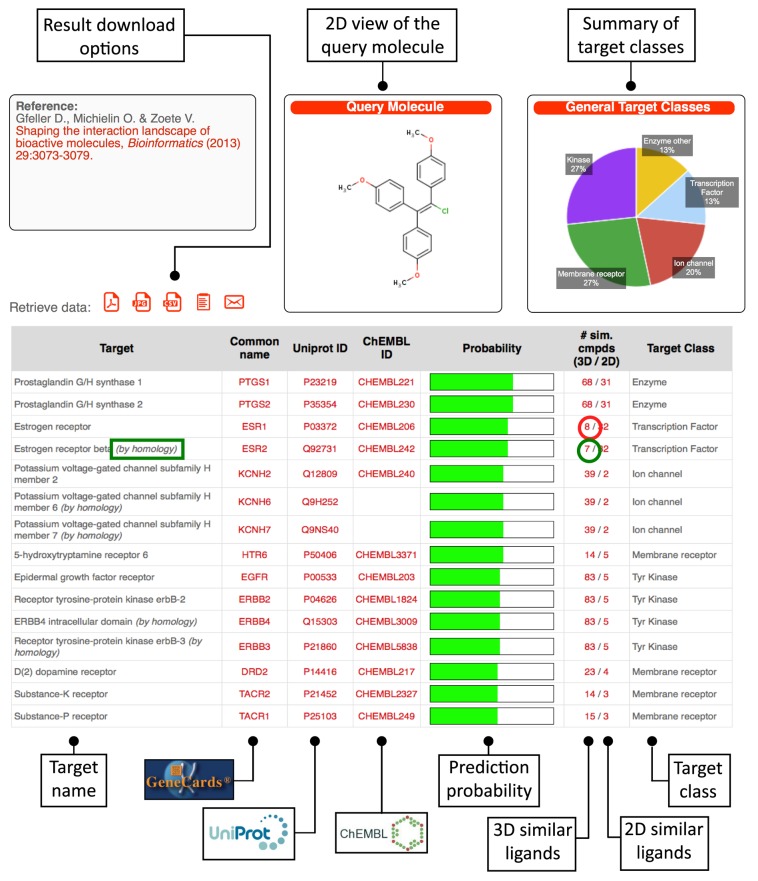
Prediction result page. This page shows the list of predicted targets for the query molecule (here chlorotrianisene). Targets are ranked according to their scores. Links to GeneCards (under ‘Common name’ column), UniProt and ChEMBL (when available) are provided. Green bars indicate the estimated probability of a protein to be a true target given its score. The sixth column (# sim cmpds 3D/2D) shows the number of ligands of the predicted target or its homologs that display similarity with the query molecule based on either 2D or 3D similarity measures. These numbers are linked to pages containing information about these ligands. For instance, the number circled in red provides a link to the list of ligands of ESR1 or its homologous proteins that display similarity with the query molecule (see Figure 2A). The pie chart shows the distribution of target classes. Predictions based on homology are indicated with ‘(by homology)’ (see the green box).

The result page lists the predicted targets with their common name together with links to GeneCards (30) (for human proteins), UniProt (31) and ChEMBL (6) databases when available (see Figure 1). Targets are ranked according to their score with respect to the query molecule. The target classes are displayed in the last column. These classes were retrieved from the ChEMBL target annotation and in general correspond to the *l*1 level in the target classification (6). Exceptions include enzymes and transcription factors for which more detailed classification based on *l*2 or *l*3 levels is sometimes shown if they occur frequently in the target list (e.g. Tyr kinase, see Figure 1). The pie chart on the top right of the page shows a summary of the different target classes present among the predicted targets. All results can be downloaded as text (.txt or .csv), images (.jpg), printable report (.pdf), copied to the clipboard or sent to an email address by clicking on the links following the ‘Retrieve data:’ field. The probability derived from the target scores (see Supplementary Materials) is displayed in the fifth column as a horizontal bar (see Figure 1).

In the example of Figure 1, the predicted targets of chlorotrianisene (CHEMBL1200761) include Prostaglandin G/H synthase 1 (COX-1) and estrogen receptor (ESR1). Chlorotrianisene is a known inhibitor of COX-1 (13), although the interaction is not present in ChEMBL. Moreover, while no direct binding between chlorotrianisene and estrogen receptor is reported in ChEMBL, functional assay results in this database indicate that chlorotrianisene is active on estrogen receptor (32). These results show that, in this case, several of the predictions are true-positives.

**Figure 2.**
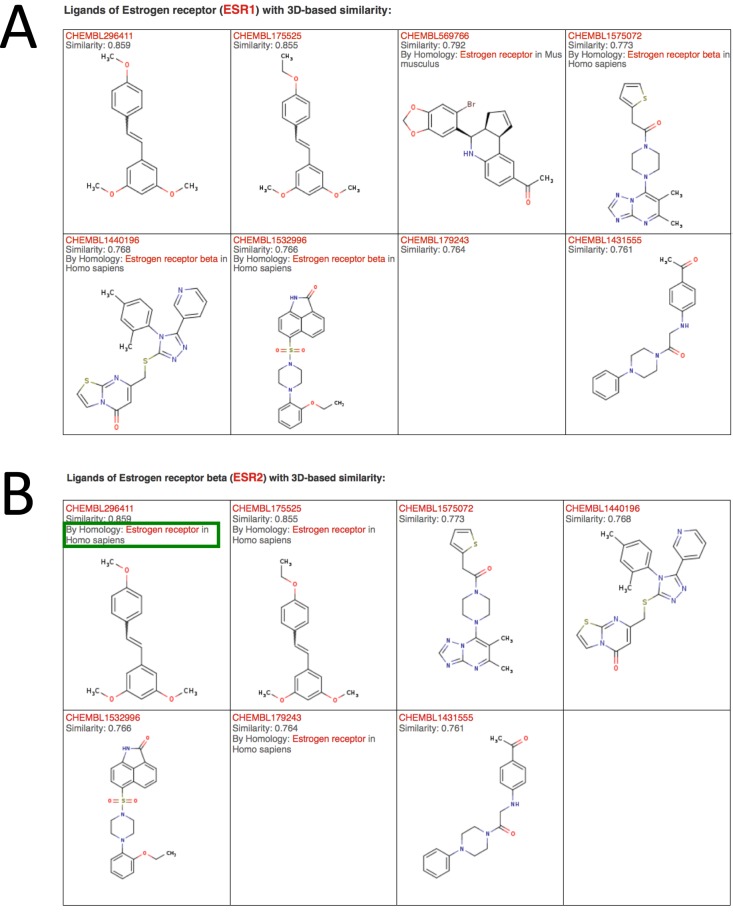
(**A**) List of ligands of ESR1 or its homologous proteins displaying 3D similarity with a query molecule (here chlorotrianisene). This page is obtained by following the link in the red circle in Figure 1. Molecules are ordered based on their 3D similarity with the query molecule. (**B**) List of ligands of ESR2 or its homologous proteins displaying similarity with a query molecule (here chlorotrianisene) obtained by following the link in the green circle in Figure 1. If a molecule is a ligand of a homologous protein of the predicted target, the actual target as well as its organism is indicated (see the green box). When the most similar molecule is a ligand of a homologous protein, the prediction is labeled as ‘by homology’ in the result page (Figure 1). A link to the ChEMBL entry is provided for each compound.

To enable users to visually explore the ligands of the predicted targets, all ligands with a similarity (either 2D or 3D) larger than a minimal threshold value can be examined by following the links provided in the sixth column. Figure 2A shows an example of the results obtained by following the link in the red circle of Figure 1. Ligands are listed according to their similarity with the query molecule. A threshold for 3D similarity values has been set to 0.75 and the one for 2D similarity values to 0.45. Below these thresholds, ligands show very low similarity with the query molecule and are not listed. A link to the ChEMBL entries is provided for the ligands and the similarity with the query molecule is indicated. We note that manually exploring the ligands similar to the query molecule is strongly recommended to assess how reasonable the predictions are and to see what kind of ligands display the strongest similarity with the query molecule.

Finally, help pages with interactive screenshots of the website are available, an FAQ page is provided to guide users, and some of the raw data used in the predictions can be retrieved via the download page.

## HOMOLOGY-BASED PREDICTIONS

Proteins originating from a common ancestor in general display a high degree of sequence and structure similarity. From a computational point of view, this similarity has been widely used in protein structure and function prediction, for instance (33,34). Recently, it has been shown that the binding of small molecules is also often conserved between homologs (35–37). In particular, orthologous proteins in close species such as human and rat often share most of their ligands (36). The same holds for paralogs, although the degree of similarity between ligands of paralogous proteins is slightly lower than between orthologous proteins (36).

In SwissTargetPrediction, we provide the possibility to map predictions based on protein homology, both within and between organisms. Orthologs and paralogs were retrieved from Ensembl Compara (38), Treefam (39) and orthoDB (40), using the union of all three datasets. Homology-based predictions were carried out as follows: the query molecule is compared to all molecules that bind to targets that have homology with a protein in the selected organism. Predictions are then carried out as if the ligands of these proteins were actual ligands of their homologs in the selected organism. If the ligand most similar to the query molecule is only observed to bind to a homologous protein, predictions are listed as ‘by homology’ on the SwissTargetPrediction result page (see Figure 1, green box). Moreover, in the list of ligands similar to the query molecule, those binding only to homologous targets are also designated with ‘By Homology’ and the actual target is indicated (Figure 2B, green box). For instance, in Figure 1 chlorotrianisene (CHEMBL1200761) is predicted to bind ESR2 mainly because it shows similarity with ligands of ESR1 (see Figure 2A). The predicted target ESR2 is therefore annotated with ‘by homology’ (green box, Figure 1). Figure 2B shows the list of most similar ligands obtained by following the link in the green circle of Figure 1. As the most similar molecule is a ligand of ESR1, it is labeled with ‘By homology’ and both the actual target and the organism are displayed. We note that for organisms with less data (e.g. horse, cow), many predictions might be based on homology with targets in other species.

Including homology-based predictions allowed us to expand the list of predicted targets from 2686 to over 12 188 in all five organisms studied here (see Table 1). As some of these proteins do not have reported bioactivity data directly associated with them, they may not be in the ChEMBL database. This is the reason why for instance KCNH6 and KCNH7 do not have ChEMBL IDs in Figure 1. Homology relationships between all targets can be downloaded at http://www.swisstargetprediction.ch/download.php.

## VALIDATION DATASET

Extensive cross-validation of the SwissTargetPrediction algorithm has been published previously (18). To complement these data, we also tested our method against a new set of molecules that are not present in the training set. In particular, we used molecules from version 17 of ChEMBL (6) that were not present in version 16 (i.e. not present in the training set). We further required that each molecule be involved in at least one positive (<2 μM) and one negative (>50 μM) interaction. This resulted in a set of 213 molecules with 346 positive and 278 negative interactions. To obtain a more balanced dataset that better reflects the much larger number of non-interacting protein–ligand pairs, we included additional negative interactions by linking the molecules in our test set to randomly chosen targets present in ChEMBL (version 16) so as to have five times more negative than positive interactions for each molecule. The full benchmark dataset can be downloaded on our website (http://www.swisstargetprediction.ch/download.php). We then ran the SwissTargetPrediction algorithm as implemented on the website to assess how accurate the predictions are. This resulted in an average AUC value of 0.87 on this external test set of both positive and negative interactions. We also assessed how often the known targets fall into the top predicted ones in the SwissTargetPrediction general output (see Figure 1). For 70% of the ligands, at least one of the known targets is found among the first 15 top predicted ones and for 31% of the ligands in our test set, the best predicted target is a true-positive. For instance, molecule CHEMBL2325087 (SMILES: NC(=S)N1N=C(CC1c1ccc2ccccc2c1)c1ccc(Cl)c(Cl)c1) binds to EGFR and ERBB2 with sub-micromolar activity (41) and these two targets are accurately predicted by SwissTargetPrediction (see Supplementary Figure S2). Although we cannot exclude that some molecules in our test set were actually developed based on their similarity with known ligands, our results strongly indicate that SwissTargetPrediction provides reliable predictions that can be used in follow-up experiments.

## DISCUSSION

SwissTargetPrediction has been primarily developed for identifying targets of molecules known to be bioactive. Nevertheless, users can upload any small molecule, real or virtual, even without prior knowledge of its potential effects. In this case, the predicted targets may be relevant, especially if the similarity with known ligands is high. The predictions may also provide hints on how a compound or a scaffold might be chemically modified in order to increase its activity on a given target by comparing with known ligands that share some similarity (see also (42)). However, we point out that prediction accuracy is expected to be significantly lower for molecules with unknown bioactivity. This can be understood by noting that SwissTargetPrediction will always suggest some target, based on the assumption that if the molecule is active, it will likely bind to some protein. For molecules with unknown bioactivity, this assumption is not valid per se and the molecule may not bind to any protein, in which case all predicted targets are false-positives. In particular, inactive compounds can sometimes exhibit good similarity with active molecules if they have been obtained by modifying an active compound at some key position that was crucial for its interactions. This is a known limitation of ligand-based approaches when applied to any kind of compounds and therefore target predictions should be interpreted with care in the absence of indication of bioactivity.

Homology-based mapping of target predictions is increasingly being recognized as a powerful approach to translate results obtained in model organisms to human (35,36,43). In this work, we have considered homology relationships between and within five vertebrate species, for which most homologous proteins display a very high sequence identity and similar functions. Therefore, we did not filter out any homology relationship. For more distant organisms (e.g. worm or yeast), greater care should be taken, for instance by allowing only mapping between orthologous proteins that have conserved binding sites or high overall sequence identity. Another possible issue with homology-based mapping arises with molecules that are specifically designed to target some members of a protein family and not others. Our algorithm, as most other ligand-based methods, will likely fail to detect these subtle differences. For instance, in Supplementary Figure S2, molecule CHEMBL2325087 is also predicted to bind to ERBB3 with equal probability, although the experimental activity (51 μM) is much lower than for EGFR and ERBB2(41). To address such issues, one possibility is to use other orthogonal computational approaches, such as structure-based analyses or molecular docking (44,45), to refine the predictions by considering small changes in protein binding sites that could confer specificity to some targets.

In SwissTargetPrediction, we use a probability derived from our cross-validation analysis to rank the targets and estimate the accuracy of the predictions. Other approaches have been proposed to assess the confidence of predictions. For instance, in Keiser *et al.* (1), an E-value is computed from the 2D similarity with the set of ligands of a target. This E-value is derived from the statistics of similarity values with all ligands (above a certain threshold), while in our case only the most similar ligand according to each similarity measure is considered. Our probabilities can be interpreted in terms of precision (i.e. number of true-positives divided by the number of predicted targets), while E-values indicate how likely it would be to find a molecule with a given average similarity to the set of ligands of a target. In practice, the most similar ligands are those contributing most to the E-value, so the two approaches are not necessarily fundamentally different. Also, predictions with very low probability in our approach correspond to low similarity values, and therefore would result in high E-values. Importantly, we point out that, by combining different kinds of chemical similarity measures, our approach can explore more diverse regions of the chemical space (18).

## CONCLUSION AND OUTLOOK

SwissTargetPrediction is part of an important initiative of the Swiss Institute of Bioinformatics to provide online tools for computer-aided drug design, many of which are already available (42,44,46–48). In future developments, SwissTargetPrediction will be further integrated with these tools, for instance by predicting potential binding modes with SwissDock (44). Moreover, as large screening campaigns are increasingly being carried out in different organisms both in industry and academia (49,50), SwissTargetPrediction will be regularly updated and new organisms added to it. This will enable users to efficiently harness the wealth of publicly available data to accurately predict new targets for bioactive small molecules in diverse species.

## SUPPLEMENTARY DATA

Supplementary Data are available at NAR Online, including references [1–6].

Supplementary Data
