# Supplementary material for: SwissTargetPrediction: a web server for target prediction of bioactive small molecules
Source: Nucleic Acids Res. 2014 May 3;42(Web Server issue):W32–8. doi: 10.1093/nar/gku293 (PMC4086140; doi:10.1093/nar/gku293)
Supplement: Supplementary Data [file supp_42_W1_W32__index.html]

Supplementary Data 

# SwissTargetPrediction: a web server for target prediction of bioactive small molecules

## Supplementary Data

**Files in this Data Supplement:**

- SUPPLEMENTARY DATA
