# Supplementary material for: SwissTargetPrediction: a web server for target prediction of bioactive small molecules
Source: Nucleic Acids Res. 2014 May 3;42(Web Server issue):W32–8. doi: 10.1093/nar/gku293 (PMC4086140; doi:10.1093/nar/gku293)
Supplement: Supplementary Data [file supp_gku293_nar-00231-web-b-2014-File004.doc]

**Supplementary Material**

**SwissTargetPrediction: a web server for target prediction of bioactive small molecules**

**Authors**: David Gfeller^1^, Aurélien Grosdidier^1^, Matthias Wirth^1^, Antoine Daina^1^, Olivier Michielin^1,2,3*^, Vincent Zoete^1*^

^1^Swiss Institute of Bioinformatics (SIB), Quartier Sorge, Bâtiment Génopode, CH-1015 Lausanne, Switzerland.

^2^Ludwig Institute for Cancer Research, Centre Hospitalier Universitaire Vaudois, Lausanne, Switzerland

^3^Department of Oncology, Centre Hospitalier Universitaire Vaudois, Lausanne, Switzerland

**1. Computing the target scores**

For a given query molecule, the 2D (based on FP2 fingerprints in OpenBabel) and 3D (based on Electroshape (1)) similarity values are computed against all 280,381 molecules used in SwissTargetPrediction, as described in (2). The 2D approach compares fingerprints describing each molecule and similarity is computed as the Tanimoto coefficient. In the 3D approach, molecules are represented by an 18-dimensional vector (1). The Manhattan distance (*d_ij_*) between these vectors is used to compute distances and the similarity between two vectors is given by $1/\left(1-\frac{1}{18}d_{ij}\right)$ (see (1,2)). As molecules can adopt different conformations, up to 20 conformations have been generated for each molecule. The final 3D similarity value between two molecules corresponds to the highest value of the 400 (20x20) pairwise combinations of all possible conformations (3). The different 3D conformations are generated from the inputted SMILES using the ChemAxon molconvert tool, which is based on the Dreiding force field (4). By default, the 20 lowest energy conformers were selected. For molecules with less than 20 possible conformers, all conformers generated by molconvert were considered. Protonation state is determined at pH7.4 using OpenBabel. Atomic partial charges are determined with the ChemAxon cxcalc tool (version 5.3.1). Atomic contributions to molecular lipophilicity are taken from the Wildman and Crippen fragmental system (5).

For each target, the most similar ligand according to each similarity measure is retrieved. The two similarity values *s’_1_* and *s’_2_,* with *s’_1_* corresponding to the 3D similarity and *s’_2_* to the 2D similarity, are then normalized. We point out that in practice only similarity values *s’_1_*>0.65 and *s’_2_*>0.3 have been computed. These similarity values are normalized: *s_1_*=(*s’_1_*-0.65)/(1-0.65) and *s_2_*=(*s’_2_*-0.3)/(1-0.3). They are then combined using a logistic regression: *f*(*s_1_, s_2_*)*=*(*1+*exp[*-a_0_-a_1_s_1_-a_2_s_2_*])*^-1^*. Coefficients for the logistic regression are listed in Table S1 and have been calculated as described in (2). It should be noted that these coefficients differ for different ligand sizes, as it was observed that optimal combinations change with ligand size (2). The final target scores take values between 0 and 1, with the largest value corresponding to targets with ligands most similar to the query molecule. These scores have been used to estimate the precision of the method (see below).

**2. Determining the probability of the predictions**

All molecules with human targets used in SwissTargetPrediction have been grouped according to their size. For each group, a random subset of 1,000 molecules has been selected to evaluate the precision of the predictions. For each of these molecules, target prediction was carried out by allowing comparison with all other ligands, except the molecule itself (leave-one-out cross-validation). Then precision curves were calculated by computing the number of true positives divided by the number of predicted targets over all molecules of the same size. As these curves are not always increasing due to low sampling for the highest-ranking targets, the final precision curves were estimated by fitting a monotonically increasing function (in this case a logistic function) to the experimental values. The fitted curves were then used to map the target scores to the probability values used in Figure 1. It is important to note that these probabilities are given as a general indication of the expected accuracy of the predictions. Since they are derived from a cross-validation analysis, some internal biases of the training data could result in slightly over-estimating this expected probability. Moreover, these probabilities should not be compared for different molecules, but only used to rank the predicted targets for a given query molecule.

**Supplementary Figures**

**Figure S1:** Screenshot of the homepage of SwissTargetPrediction. Molecules can be inputted as SMILES or drawn in 2D using the graphical interface. Predictions can be carried out in five different organisms.

**Figure S2:** Target prediction for small molecule CHEMBL2325087 (SMILES: NC(=S)N1N=C(CC1c1ccc2ccccc2c1)c1ccc(Cl)c(Cl)c1). **A**: 2D structure of the small molecule. **B**: list of predicted targets. Affinity values are not part of the web site and are shown for the discussion. They have been retrieved from (6).

**Supplementary Tables**

**Table S1**: List of logistic regression coefficients for different molecule sizes (see main text).

| **Number of heavy atoms** | **a_0_** | **a_1_** | **a_2_** |
| --- | --- | --- | --- |
| <=10 | -3.262413174 | 6.322854779 | 6.568394002 |
| 11 | -3.72535153 | 5.095483542 | 6.417589941 |
| 12 | -3.837945832 | 4.912160662 | 6.662162587 |
| 13 | -4.165145117 | 5.434698827 | 6.386047615 |
| 14 | -4.098154884 | 5.071728873 | 5.880153979 |
| 15 | -4.329105882 | 4.856611016 | 5.78062749 |
| 16 | -4.315410702 | 4.48602273 | 5.757473315 |
| 17 | -4.748260105 | 4.821008953 | 6.427766956 |
| 18 | -4.661329474 | 4.64756482 | 6.02228924 |
| 19 | -4.771876103 | 4.197614059 | 6.603873079 |
| 20 | -4.876246496 | 4.132595229 | 6.827145916 |
| 21 | -4.857226952 | 3.981143488 | 6.885403177 |
| 22 | -5.095908342 | 3.929515519 | 7.271275952 |
| 23 | -5.292956579 | 4.209571935 | 7.351816261 |
| 24 | -5.548725696 | 4.578577348 | 7.440540621 |
| 25 | -5.407423393 | 4.291511774 | 7.337653287 |
| 26 | -5.61563412 | 4.295384968 | 7.705961587 |
| 27 | -5.575990482 | 4.147153936 | 7.633867426 |
| 28 | -5.643197331 | 4.092921412 | 8.124714371 |
| 29 | -5.562423769 | 3.872254267 | 8.185273689 |
| 30 | -6.187130962 | 4.399171853 | 8.587322882 |
| 31 | -6.492001657 | 5.008318936 | 8.523311093 |
| 32 | -6.554879989 | 4.970726497 | 9.181886497 |
| 33 | -6.482603651 | 4.077656396 | 10.11025478 |
| 34 | -5.83552547 | 3.269588783 | 9.218469067 |
| 35 | -6.824822923 | 4.286491756 | 10.17063931 |
| 36 | -6.542919592 | 4.362283659 | 9.659730248 |
| 37 | -7.434668954 | 4.842267054 | 11.44600355 |
| 38 | -6.768343188 | 4.793564549 | 9.528935047 |
| 39 | -6.796201259 | 4.465436795 | 10.05670984 |
| 40 | -6.64318689 | 3.762519169 | 10.40374227 |
| 41 | -6.634017017 | 3.730771109 | 10.38115996 |
| 42 | -6.489778456 | 3.506693437 | 10.03737076 |
| 43 | -6.384341828 | 2.11617237 | 11.48422777 |
| 44 | -6.440368553 | 4.099489258 | 9.536395503 |
| 45 | -7.032830335 | 4.462265541 | 10.10085302 |
| 46 | -5.755040849 | 3.633312152 | 8.432120752 |
| 47 | -6.86172496 | 3.561497186 | 10.65099107 |
| 48 | -6.866811123 | 3.269922323 | 10.84633127 |
| 49 | -6.236246869 | 3.226544243 | 9.893332871 |
| 50 | -6.432453649 | 3.41102254 | 10.12041471 |
| 51 | -6.150707677 | 3.123486059 | 9.53250525 |
| 52 | -5.824680261 | 2.51544752 | 9.720749664 |
| 53 | -5.891579097 | 2.09484265 | 9.468930979 |
| 54 | -5.369860942 | 2.981799311 | 7.472380926 |
| 55 | -5.396801163 | 2.757740456 | 7.721935419 |
| 56 | -5.107974444 | 3.045529243 | 7.214145703 |
| 57 | -5.164998429 | 2.379775135 | 7.777750138 |
| 58 | -5.510935556 | 2.887713559 | 7.774578777 |
| 59 | -5.904099676 | 4.120870416 | 7.877062122 |
| >=60 | -5.628526189 | 2.904582753 | 7.503525186 |

**References**

1. Armstrong, M.S., Finn, P.W., Morris, G.M. and Richards, W.G. (2011) Improving the accuracy of ultrafast ligand-based screening: incorporating lipophilicity into ElectroShape as an extra dimension. *J Comput Aided Mol Des*, 25, 785-790.

2. Gfeller, D., Michielin, O. and Zoete, V. (2013) Shaping the interaction landscape of bioactive molecules. *Bioinformatics*, 29, 3073-3079.

3. Gfeller, D., Michielin, O. and Zoete, V. (2013) SwissSidechain: a molecular and structural database of non-natural sidechains. *Nucleic Acids Res*, 41, D327-D332.

4. Mayo, S.L., Olafson, B.D. and Goddard, W.A. (1990) DREIDING: a generic force field for molecular simulations. *J. Phys. Chem*, 94, 8897-8909.

5. Wildman, S.A. and Crippen, G.M. (1999) Prediction of Physicochemical Parameters by Atomic Contributions. *J Chem Inf Model*, 39, 868–873.

6. Yang, W., Hu, Y., Yang, Y.S., Zhang, F., Zhang, Y.B., Wang, X.L., Tang, J.F., Zhong, W.Q. and Zhu, H.L. (2013) Design, modification and 3D QSAR studies of novel naphthalin-containing pyrazoline derivatives with/without thiourea skeleton as anticancer agents. *Bioorganic & medicinal chemistry*, 21, 1050-1063.
